# Modeling Consumer Adoption of Digital Mental Health Services (DMHS) in Indonesia: The Role of Desire

**DOI:** 10.12688/f1000research.182672.2

**Published:** 2026-10-26

**Authors:** Mochammad Riyadh Rizky Adam, Oyyappan Duraipandi, Derek Ong Lai Teik, Wenny C. Mandagie

**Affiliations:** 1 Business and Management, Lincoln University College, Petaling Jaya, Selangor, 47301, Malaysia; 2 Management, University of Jakarta International, West Jakarta, Jakarta, 11480, Indonesia; 3 Marketing, University of Hertfordshire Hertfordshire Business School, Hatfield, England, UK

**Keywords:** digital mental health services, health marketing, HBM, MGB

## Abstract

**Introduction:**

The rapid growth of digital mental health services (DMHS) offers new opportunities to address unmet mental health needs, yet consumer adoption remains uneven, particularly in emerging economies.

**Objectives:**

Drawing on the model of goal-directed behavior (MGB) and the health belief model (HBM), this study develops and tests an integrated framework that positions desire to manage mental health as a central motivational mechanism underlying consumers’ intention to purchase DMHS in Indonesia.

**Methods:**

The study employed a quantitative research method, a self-administered online survey, among Indonesians aged 18–45 who had engaged in online shopping within the previous 6 months. Screening questions were used to assess the respondent’s eligibility. The DAS-21 (depression-anxiety-stress) scales were used to measure the respondents’ mental health condition with their consent. The PLS-SEM with SmartPLS was used to examine the proposed relationships.

**Result:**

The attitude toward purchasing DMHS and the subjective norm significantly influence desire, while perceived severity and perceived susceptibility also emerge as important motivational antecedents. Perceived behavioral control does not significantly affect desire, suggesting that instrumental capability alone is insufficient to activate emotional motivation in the mental health context. Importantly, desire exhibits a strong and positive effect on behavioral intention, confirming its role as the most proximal predictor of adoption intention.

**Conclusion:**

The study aimed to model consumer adoption of digital mental health services (DMHS) in Indonesia by integrating the goal-directed behavior (MGB) model with the health belief model (HBM), positioning the desire to manage mental health as a central motivational mechanism. The findings claim there is robust evidence that desire plays a pivotal role in translating cognitive evaluations and health beliefs into behavioral intention to purchase DMHS.

## Introduction

Mental health has become an increasingly urgent public health concern, driven by accelerated urban development, mounting socioeconomic pressures, and the enduring consequences of the SARS-CoV-2 pandemic. Simultaneously, advances in digital technology have transformed the healthcare landscape by facilitating the emergence of digital mental health services (DMHS), which offer scalable, accessible, and relatively affordable alternatives for psychological support ( Schueller et al., 2019). Despite their potential to address gaps in conventional mental healthcare delivery, the uptake of these services remains uneven, particularly across emerging economies such as Indonesia. While digital health adoption has attracted growing scholarly attention, empirical research examining the behavioral processes underlying consumer adoption decisions toward DMHS remains limited.

To better understand these behavioral mechanisms, this study adopts the model of goal-directed behavior (MGB) as its primary theoretical foundation. Originally proposed by Perugini and Bagozzi (2001) as an extension of the theory of planned behavior (TPB), the MGB introduces desire as a central motivational construct linking cognitive evaluations—including attitude, subjective norm, and perceived behavioral control—to behavioral intention. By explicitly incorporating motivational and affective dimensions, the model offers a broader explanatory perspective than traditional rational-choice frameworks, making it particularly suitable for investigating technology-related decision-making in emerging service contexts. Although the MGB has demonstrated explanatory power across diverse domains ( Thomas-Francois et al., 2023; Poon & Tung, 2022), its application to digital mental health consumption remains relatively underdeveloped, particularly in relation to the psychological and economic factors shaping consumer adoption behavior.

Building on perspectives from behavioral marketing and health economics, this study extends the MGB by integrating two constructs derived from the health belief model (HBM), namely perceived severity and perceived susceptibility. These constructs capture individuals’ perceptions of health-related threats and are introduced to reflect the extent to which perceived mental health risks act as motivational drivers of behavioral intention. The underlying premise is that individuals who recognize the potential personal, social, and economic consequences of unmanaged mental health conditions may demonstrate stronger motivation to seek digital mental health solutions.

MGB proposes that attitude, subjective norm, and perceived behavioral control shape intention primarily through desire, which represents the motivational state that transforms evaluations into goal pursuit ( Perugini & Bagozzi, 2001). Accordingly, favorable evaluations of DMHS, perceived approval from significant others, and perceived capability to access the service were expected to strengthen the desire to manage mental health. Extending MGB with HBM, perceived severity and susceptibility were expected to heighten the personal relevance of mental-health threats and thereby strengthen desire for protective action. Prior studies support these pathways across mHealth and digital-service contexts ( Schuster et al., 2015; Vishwakarma et al., 2019; Poon & Tung, 2022; Thomas-Francois et al., 2023).

Situated within the Indonesian market, this research investigates the behavioral determinants influencing consumers’ intention to purchase DMHS in one of Southeast Asia’s fastest-growing digital economies. Indonesia provides a particularly relevant empirical setting due to its expanding digital infrastructure, rising awareness of mental health issues, and increasing consumer engagement with online services. By combining behavioral theory with economic and technological perspectives, this study seeks to clarify how psychological motivations are translated into market demand for digital health innovation. Accordingly, this study addresses the following research question: how do the core variables of the model of goal-directed behavior, together with perceived severity and perceived susceptibility, influence Indonesian consumers’ intention to adopt digital mental health services, and what implications do these relationships hold for the sustainable development of the digital health sector?

This research contributes to literature in three important ways. First, it advances theoretical understanding by extending the explanatory scope of the MGB through the incorporation of health belief constructs as presented in Figure 1, thereby offering a more comprehensive framework for examining consumer behavior in mental health service adoption. Second, it provides practical insights for digital health providers and industry stakeholders by identifying the psychological mechanisms that influence consumers’ willingness to engage with DMHS. Third, from a broader economic perspective, the study positions DMHS adoption within the context of the knowledge-driven digital economy. By demonstrating how psychological readiness shapes behavioral intention and market participation, the findings offer valuable implications for policymakers and practitioners seeking to foster innovation, investment, and inclusive growth within the digital health ecosystem.

**Figure 1.**
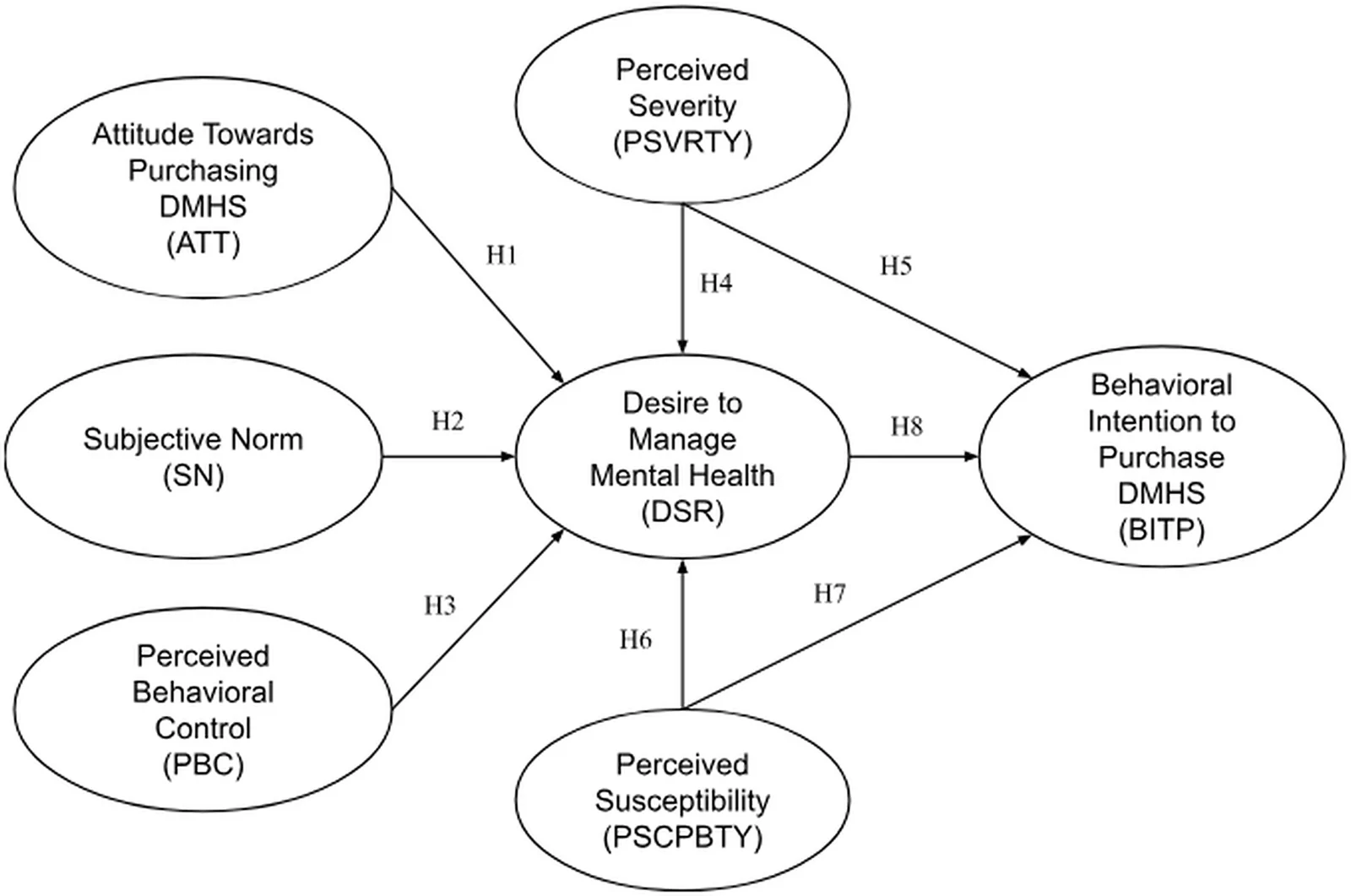
Proposed conceptual framework of the study.

## Methods

### Research context

The empirical investigation was conducted primarily within the Greater Jakarta metropolitan region (Jabodetabek), which includes Jakarta, Bogor, Depok, Tangerang, and Bekasi, while also incorporating respondents from other regions across Indonesia. This area was selected because it represents the country’s principal economic and technological center, characterized by relatively advanced digital infrastructure, widespread internet access, and high levels of engagement with online services. The study was implemented over an eight-month period, from May to December 2025. The research process was organized into several sequential phases. Instrument development and refinement were completed in May 2025, followed by ethics review and institutional approval in June 2025. Data collection took place between July and October 2025. Statistical analysis was conducted during November 2025, while the interpretation of findings and preparation of the initial manuscript were finalized in December 2025.

### Research design

This study adopted a quantitative cross-sectional research design to examine the determinants influencing consumers’ intention to purchase digital mental health services (DMHS). A quantitative approach was considered appropriate because it enables the statistical examination of relationships among multiple latent constructs and facilitates objective assessment of behavioral patterns across a relatively large sample ( Lo et al., 2020). The cross-sectional design was selected because the study sought to capture consumers’ behavioral perceptions and intentions at a specific point in time rather than observe behavioral changes across multiple periods ( Wang & Cheng, 2020). Ethical clearance for this research was granted by the Research & Innovation division of Lincoln University College under approval number LUC/MKT/IND/SP/007/310.

### Population and sampling procedure

The target population consisted of Indonesian consumers aged 18 to 45 years who had engaged in at least one form of online purchasing activity within the six months preceding data collection. Eligible digital transactions included purchases made through e-commerce marketplaces, social commerce platforms, and app-based food and beverage services. This age range was selected because prior evidence indicates that individuals within this demographic represent the most active segment of Indonesia’s digital ecosystem, demonstrating high levels of internet use, smartphone adoption, and participation in digital transactions ( Lautania & Dinaroe, 2024; Siahaan et al., 2022). Their familiarity with online purchasing behavior makes them particularly relevant for investigating adoption intention toward digital health services. A non-probability sampling strategy was employed, combining purposive sampling with snowball recruitment through online channels. Eligibility screening was conducted at the beginning of the survey to verify respondents’ age, recent online purchasing experience, and familiarity with digital platforms. This screening process ensured that the final sample reflected individuals with sufficient digital exposure to evaluate DMHS meaningfully.

### Data collection procedure

Primary data were gathered through a self-administered online questionnaire hosted on Google Forms. Prior to participation, respondents were presented with an informed consent statement outlining the study’s objectives, confidentiality assurances, the voluntary nature of participation, and the right to withdraw from the study at any time. Electronic informed consent was obtained from all participants before they proceeded to the questionnaire. Participants who did not provide consent were automatically excluded from further participation and could not access the subsequent sections of the survey, including the respondent eligibility questions. Only responses from participants who provided informed consent were included in the final analysis. To broaden respondent reach and improve sample heterogeneity, the survey link was distributed through both organic sharing and paid targeted promotion across several digital platforms, including Instagram, TikTok, LinkedIn, X, Facebook, and WhatsApp. The use of multiple dissemination channels helped minimize platform-specific sampling bias while increasing geographic diversity. An online survey approach was selected due to its operational efficiency, cost-effectiveness, flexibility, and ability to reach digitally active populations across dispersed locations ( Ball, 2019). To ensure conceptual consistency across languages, the questionnaire underwent a translation and back-translation process between English and Bahasa Indonesia following Brislin’s (1970) recommended procedure.

### Sample size determination

A total of 462 valid responses were retained for analysis after screening and data cleaning. The minimum sample requirement was calculated using the 10-times rule commonly applied in Partial Least Squares Structural Equation Modeling (PLS-SEM), which recommends that the sample size should be at least ten times the largest number of structural paths directed at any endogenous construct ( Barclay et al., 1995). Based on the proposed structural model, the minimum required sample was 50 observations. The sample achieved substantially exceeded this threshold, thereby providing adequate statistical power for parameter estimation and hypothesis testing ( Hair et al., 2022).

### Measurement of constructs

All latent variables were operationalized using multi-item scales adapted from previously validated studies to ensure content validity and conceptual consistency. Multi-item measurement was preferred over single-item indicators due to its superior reliability and ability to capture construct complexity ( Churchill, 1979). Responses were recorded using a five-point Likert scale ranging from 1 (strongly disagree) to 5 (strongly agree). The measurement scales were adapted as follows:

• Attitude toward purchasing DMHS: six items adapted from Vishwakarma et al. (2019), Haldar and Goel (2019), and Thomas-Francois et al. (2023)
• Subjective norm: five items adapted from Schuster et al. (2015) and Thomas-Francois et al. (2023)
• Perceived behavioral control: five items adapted from Vishwakarma et al. (2019) and Thomas-Francois et al. (2023)
• Perceived severity: seven items adapted from Daragmeh et al. (2021), Wei et al. (2021), with one additional item developed specifically for this study
• Perceived susceptibility: five items adapted from Daragmeh et al. (2021), Sun et al. (2013), supplemented by author-developed items
• Desire to manage mental health: three items adapted from Schuster et al. (2015)
• Behavioral intention to purchase DMHS: four items adapted from Schuster et al. (2015)

Before final deployment, the instrument was reviewed by three experts specializing in digital consumer behavior and psychology. Their evaluation focused on wording clarity, contextual appropriateness, and construct relevance.

### Research instrument

The questionnaire used in this study was specifically developed for the present investigation and underwent preliminary testing to establish its validity and reliability prior to full-scale distribution. The instrument consisted of six main sections: (1) an introduction to the study; (2) informed consent; (3) eligibility screening questions; (4) demographic and behavioral profiling; (5) construct measurement items; and (6) current mental health condition assessment. This structure was designed to ensure respondent eligibility while systematically capturing all variables relevant to the proposed conceptual framework.

### Data analysis technique

The hypothesized relationships were examined using Partial Least Squares Structural Equation Modeling (PLS-SEM) implemented through SmartPLS. PLS-SEM was selected for several methodological reasons. First, the proposed framework integrates constructs from both the model of goal-directed behavior (MGB) and the health belief model (HBM), resulting in a structurally complex model involving multiple simultaneous relationships and mediation pathways. Second, the study prioritizes predictive accuracy regarding behavioral intention rather than strict covariance-based model fit assessment. Third, online survey datasets frequently exhibit deviations from multivariate normality, making PLS-SEM particularly suitable due to its limited distributional assumptions ( Hair & Alamer, 2022). Additionally, because this research represents an exploratory theoretical integration of MGB and HBM within the digital mental health context, PLS-SEM offers an appropriate analytical framework for theory extension and predictive model evaluation.

## Results

A total of 462 valid responses were included in the final analysis. The demographic distribution of respondents is presented in Table 1. The respondent profile reflects a predominantly young, digitally engaged, and urban-based sample. Individuals aged between 18 and 35 years represented more than four-fifths of the total participants, which is consistent with evidence suggesting that younger populations demonstrate higher engagement with digital consumption and technology-mediated health behaviors ( Pretorius et al., 2019). Female respondents accounted for 68% of the sample, aligning with prior studies indicating that women tend to show greater proactive engagement in seeking mental health information and utilizing preventive health-related services ( Demirci et al., 2021). In terms of geographic distribution, 90.9% of participants resided in urban areas, reflecting Indonesia’s concentration of digital infrastructure and online health service exposure within metropolitan centers ( IBC Team, 2024). Educational attainment was also relatively high, with 51.9% of respondents having completed tertiary education. This profile suggests sufficient digital literacy to evaluate and navigate digital mental health platforms effectively ( van Deursen & van Dijk, 2014).

**Table 1.** Respondent profiles.

Demographic variables	Item	Frequency	(%)
AGE	18–25 years	225	48.7%
	26–35 years	158	34.2%
	36–45 years	79	17.1%
GENDER	Male	147	31.8%
	Female	315	68.2%
DOMICILE	Jakarta	130	28.1%
	Bogor	61	13.2%
	Depok	50	10.8%
	Tangerang	103	22.3%
	Bekasi	55	11.9%
	Non-Jabodetabek	63	13.6%
AREA OF RESIDENCE	Urban area	420	90.9%
	Rural area	42	9.1%
OCCUPATION	University students	100	21.6%
	Full-time employee	143	31.0%
	Freelancer	54	11.7%
	Civil servant	20	4.3%
	Entrepreneur	60	13.0%
	Homemaker	17	3.7%
	Professional (Physician, professor, lawyer, notary, etc.)	16	3.5%
	Others (internship, part-time, unemployed, etc.)	52	11.3%
THE COMPLETED HIGHEST EDUCATION	High school or equivalent	163	35.3%
	Diploma	29	6.3%
	Bachelor’s degree	240	51.9%
	Master’s degree	24	5.2%
	Doctorate	6	1.3%
MONTHLY INCOME (IDR)	< Rp 2.500.000	105	22.7%
	Rp 2.500.000 - Rp 5.000.000	105	22.7%
	Rp 5.000.001 - Rp 10.000.000	85	18.4%
	Rp 10.000.001 - Rp 20.000.000	39	8.4%
	> Rp 20.000.000	24	5.2%
	I prefer not to answer	104	22.5%
DMHS PURCHASE OR USING EXPERIENCE FROM ANY BRAND	Yes	234	50.6%
	No	228	49.4%
DMHS AWARENESS	Yes	351	76.0%
	No	111	24.0%
ONLINE SHOPPING FREQUENCY	Once a year	17	3.7%
	3–4 times a year	59	12.8%
	Once every 2 months	84	18.2%
	Once a month	126	27.3%
	More than once a month	176	38.1%
HAVE EVER PURCHASED OR ORDERED ANY HEALTH SERVICES DIGITALLY IN THE PAST ONE YEAR	Yes	255	55.2%
	No	207	44.8%
SELF-CLAIMED PERSONALITY	Introvert	302	65.4%
	Extrovert	160	34.6%
SEVERITY OF SELF-CLAIMED MENTAL HEALTH CONDITION (DAS-21)	Normal	140	30.3%
	Mild	49	10.6%
	Moderate	105	22.7%
	Severe	57	12.3%
	Extremely severe	111	24.0%

Although awareness of digital mental health services was relatively widespread, with 76.0% of respondents indicating familiarity with DMHS, only 50.6% reported previous purchase experience. This discrepancy suggests the existence of a notable awareness-to-adoption gap within the Indonesian market. Furthermore, nearly 60% of respondents reported experiencing at least moderate psychological distress, indicating meaningful latent demand for accessible digital mental health interventions. Overall, the sample characteristics are appropriate for examining behavioral intention toward DMHS adoption, as they reflect the demographic groups most commonly associated with digital health engagement globally, namely younger, urban, and educated consumers ( Pretorius et al., 2019; van Deursen & van Dijk, 2014).

Descriptive statistics further indicate moderately positive consumer perceptions toward DMHS. Respondents reported a moderate attitude toward purchasing such services ( $\bar{x}$ = 3.60), moderate perceived social support for adoption ( $\bar{x}$ = 3.38), and a relatively favorable perception of behavioral control over service usage ( $\bar{x}$ = 3.68). Perceived severity of unmanaged mental health conditions was relatively high ( $\bar{x}$ = 4.11), suggesting strong recognition of the potential consequences of neglecting mental health. Perceived susceptibility was more moderate ( $\bar{x}$ = 3.41), indicating a balanced assessment of personal vulnerability. Respondents also demonstrated a generally favorable desire to actively manage their mental health ( $\bar{x}$ = 3.88), while their intention to purchase DMHS was moderate overall ( $\bar{x}$ = 3.31).

### Measurement model assessment

The reflective measurement model was evaluated to establish internal consistency, convergent validity, and discriminant validity. Following established PLS-SEM procedures, assessment criteria included indicator loadings, composite reliability (CR), Cronbach’s alpha, and average variance extracted (AVE) ( Hair et al., 2019). Most outer loadings exceeded the recommended threshold of 0.70. A small number of indicators, including PBC5 (0.603) and PSV7 (0.687), fell slightly below this benchmark but were retained due to their conceptual relevance and acceptable contribution to construct validity ( Hair et al., 2019). Table 2 presents the reliability and convergent validity assessment of the constructs. The composite reliability values ranged from 0.867 to 0.958, while Cronbach’s alpha coefficients ranged from 0.814 to 0.945, indicating satisfactory to excellent internal consistency reliability across all constructs. Furthermore, the average variance extracted (AVE) values ranged between 0.564 and 0.841, exceeding the recommended threshold of 0.50 ( Fornell & Larcker, 1981), thereby confirming adequate convergent validity.

**Table 2.** Rho_c, Cronbach’s alpha, and AVE of the constructs.

Construct	Composite reliability (Rho_c)	Cronbach’s Alpha α	Average variance extracted (AVE)
Attitude (ATT)	0.930	0.910	0.690
Subjective norm (SN)	0.958	0.945	0.821
Perceived behavioral control (PBC)	0.867	0.814	0.568
Perceived severity (PSV)	0.900	0.871	0.564
Perceived susceptibility (PSC)	0.892	0.856	0.624
Desire (DSR)	0.941	0.906	0.841
Behavioral Intention to purchase (BITP)	0.936	0.909	0.785

Among the measured constructs, attitude toward purchasing DMHS demonstrated strong reliability (CR = 0.930; α = 0.910; AVE = 0.690), while subjective norm exhibited the highest consistency (CR = 0.958; α = 0.945; AVE = 0.821). Likewise, perceived behavioral control (CR = 0.867; α = 0.814; AVE = 0.568), perceived severity (CR = 0.900; α = 0.871; AVE = 0.564), perceived susceptibility (CR = 0.892; α = 0.856; AVE = 0.624), desire (CR = 0.941; α = 0.906; AVE = 0.841), and behavioral intention (CR = 0.936; α = 0.909; AVE = 0.785) all met established psychometric standards. These findings indicate that the measurement model satisfies the minimum reliability and convergent validity requirements recommended for PLS-SEM analysis ( Sarstedt et al., 2017).

Discriminant validity was assessed using the Fornell–Larcker criterion, heterotrait-monotrait ratio (HTMT), and cross-loading analysis. Results from the Fornell–Larcker assessment showed that the square root of each construct’s AVE exceeded its correlations with all other constructs, indicating adequate construct distinctiveness ( Fornell & Larcker, 1981). Similarly, all HTMT values were below the recommended threshold of 0.90 ( Henseler et al., 2015), further supporting discriminant validity. Cross-loading analysis confirmed that each measurement item loaded more strongly on its intended construct than on any alternative construct. No indicators displayed problematic cross-loadings exceeding 0.70 on non-target constructs. Collectively, these findings confirm satisfactory discriminant validity at both construct and indicator levels ( Chin, 1998; Hair et al., 2019).

### Structural model assessment

Following validation of the measurement model, the structural model was evaluated to test the hypothesized relationships among constructs. The assessment followed recommended PLS-SEM procedures outlined by Hair et al. (2019), including examination of collinearity diagnostics, path coefficients, and significance testing. To evaluate potential multicollinearity among predictor variables, variance inflation factor (VIF) values were examined. All inner VIF values ranged from 1.233 to 1.477, substantially below the critical threshold of 5 and comfortably within preferred ranges. These results indicate the absence of multicollinearity concerns and confirm that each exogenous construct contributed distinct explanatory value to the endogenous variables.

### Goodness of fit (GoF) and predictive performance

The adequacy of the proposed model was further assessed through multiple predictive and explanatory indicators, including R ^2^, Q ^2^, standardized root means square residual (SRMR), goodness-of-fit (GoF), PLS-Predict, and linearity diagnostics. Table 3 presents the goodness-of-fit assessment of the structural model, including the R-square, Q-square, SRMR, and GoF index values. The R-square results indicate that the model explains 28.3% of the variance in behavioral intention to purchase DMHS and 19.0% of the variance in desire to manage mental health, suggesting moderate explanatory power. In addition, the Q-square values for behavioral intention (0.231) and desire (0.164) are greater than zero, indicating that the model possesses adequate predictive relevance ( Hair et al., 2022).

**Table 3.** Goodness of fit - R square, Q square, SRMR, and GoF index.

GoF—R-square & Q-square		
	R-square	Q-square
BEHAVIORAL INTENTION TO PURCHASE DMHS	0.283	0.231
DESIRE TO MANAGE MENTAL HEALTH	0.190	0.164
**GoF—SRMR (Standardized Root Mean Square Residual)**		
	**Estimated Model**	
SRMR	0.059	
**GoF—Goodness of Fit (GoF) Index**		
**Average Communality**	**Average R-Square**	**GoF Index**
0.680	0.237	0.401

Furthermore, the SRMR value of 0.059 is below the recommended threshold of 0.08, demonstrating a good model fit ( Hair et al., 2021; Schermelleh-Engel et al. (2003). The GoF index value of 0.401 also indicates a strong overall model fit, as it exceeds the recommended cutoff value of 0.36 ( Henseler & Sarstedt, 2013; Wetzels et al., 2009), suggesting robust alignment between the empirical data and the proposed conceptual structure. Overall, these findings suggest that the proposed structural model demonstrates acceptable explanatory power, predictive relevance, and overall goodness of fit for PLS-SEM analysis.

Table 4 presents the PLS-predict assessment used to evaluate the out-of-sample predictive performance of the model. The results indicate that most PLS-SEM prediction errors (RMSE and MAE) are lower than those generated by the linear regression model (LM), suggesting that the proposed model demonstrates acceptable predictive power ( Hair et al., 2022), supporting the practical usefulness of the model for forecasting consumer adoption behavior toward DMHS. Specifically, several indicators, such as BITP2, BITP3, DSR1, DSR2, and DSR3, show lower RMSE and/or MAE values in the PLS-SEM model compared to the LM benchmark model.

**Table 4.** Goodness of fit - PLS predict.

GoF—PLS predict of behavioral intention to purchase DMHS and desire to manage mental health				
Measurement Item	PLS-SEM		LM (Linear Regression Model)	
	RMSE (Root Mean Square Error)	MAE (Mean Absolute Error)	RMSE (Root Mean Square Error)	MAE (Mean Absolute Error)
BITP1	0.874	**0.697**	0.876	0.695
BITP2	0.957	0.765	0.970	0.779
BITP3	1.021	0.810	1.028	0.815
BITP4	0.944	**0.754**	0.944	0.747
DSR1	0.904	0.728	0.932	0.742
DSR2	0.969	0.782	0.989	0.792
DSR3	0.967	0.788	0.994	0.804

Potential nonlinear relationships were examined using quadratic effect analysis in SmartPLS 4. The findings, as presented in Table 5, indicate that most structural paths were adequately represented through linear relationships. However, statistically significant quadratic effects were identified for the paths linking attitude, subjective norm, and perceived behavioral control with behavioral intention, as well as the path between perceived susceptibility and desire. These results suggest the presence of curvilinear effects, indicating that the influence of certain predictors may vary across different levels of the constructs rather than following strictly proportional linear patterns. Consistent with PLS-SEM recommendations, the inclusion of quadratic terms enhances analytical precision without compromising the validity of the primary linear structural estimates ( Hair et al., 2022).

**Table 5.** Goodness of fit - Linearity testing.

Evaluating goodness of fit (GoF)—Linearity testing (p-value should be > 0.05).			
Linearity testing	Path coefficient	P-value	Conclusion
QE (perceived severity) ⇒ behavioral intention to purchase DMHS	0.021	0.491	**Linearity is achieved**
QE (perceived severity) ⇒ desire to manage mental health	−0.068	0.057	**Linearity is achieved**
QE (desire to manage mental health) ⇒ behavioral intention to purchase DMHS	−0.052	0.100	**Linearity is achieved**
QE (perceived susceptibility) ⇒ behavioral intention to purchase DMHS	−0.064	0.126	**Linearity is achieved**
QE (perceived behavioral control) ⇒ behavioral intention to purchase DMHS	0.108	0.009	**Curvilinear relationship is present**
QE (subjective norm) ⇒ behavioral intention to purchase DMHS	−0.127	0.004	**Curvilinear relationship is present**
QE (attitude) ⇒ behavioral intention to purchase DMHS	0.105	0.006	**Curvilinear relationship is present**
QE (perceived susceptibility) ⇒ desire to manage mental health	0.089	0.019	**Curvilinear relationship is present**
QE (attitude towards purchasing DMHS) ⇒ desire to manage mental health	−0.014	0.683	**Linearity is achieved**
QE (subjective norm) ⇒ desire to manage mental health	0.046	0.243	**Linearity is achieved**
QE (perceived behavioral control) ⇒ desire to manage mental health	0.055	0.093	**Linearity is achieved**

## Discussion

The hypothesis testing results presented in Table 6 provide empirical support for five of the eight proposed relationships (p < 0.05, t > 1.96). Overall, the findings suggest that the integrated MGB–HBM framework offers meaningful explanatory power for understanding consumer intention toward digital mental health service (DMHS) adoption in Indonesia. While most motivational pathways were supported, two of the hypothesized direct relationships involving health threat perceptions and one pathway related to perceived behavioral control did not achieve statistical significance. These mixed findings offer important insights into the behavioral dynamics underlying digital mental health adoption.

**Table 6.** Hypothesis testing results.

Evaluation of structural model - Hypothesis testing (the p-value should be < 0.05)							
Proposed hypothesis	Path coefficient β	p-value < 0.05	t-value > 1.96	Confidence intervals bias-corrected		Conclusion	f-square
				2.5%	97.5%		
H1. **Attitude** towards purchasing DMHS ⇒ **desire** to manage mental health	0.166	0.002	3.102	0.062	0.271	**SUPPORTED**	0.025
H2. **Subjective norm** ⇒ **desire** to manage mental health	0.122	0.013	2.482	0.025	0.218	**SUPPORTED**	0.015
H3. **Perceived behavioral control** ⇒ **desire** to manage mental health	0.058	**0.246**	**1.159**	No need to analyze		**NOT SUPPORTED**	No need to analyze
H4. **Perceived severity** ⇒ **desire** to manage mental health	0.138	0.006	2.737	0.035	0.233	**SUPPORTED**	0.016
H5. **Perceived severity** ⇒ **behavioral intention** to purchase DMHS	−0.006	**0.899**	**0.127**	No need to analyze		**NOT SUPPORTED**	No need to analyze
H6. **Perceived susceptibility** ⇒ **desire** to manage mental health	0.156	0.004	2.908	0.049	0.257	**SUPPORTED**	0.022
H7. **Perceived susceptibility** ⇒ **behavioral intention** to purchase DMHS	0.044	**0.418**	**0.810**	No need to analyze		**NOT SUPPORTED**	No need to analyze
H8. **Desire** to manage mental health ⇒ **behavioral intention** to purchase DMHS	0.179	0.000	3.818	0.088	0.272	**SUPPORTED**	0.036

The results indicate that attitude toward purchasing DMHS positively influenced desire to manage mental health (β = 0.166, p = 0.002, t = 3.102), thereby supporting H1. This finding suggests that favorable evaluations of digital mental health services contribute to stronger motivational readiness to engage in proactive mental health management. This outcome is consistent with prior research emphasizing the central role of attitudinal evaluation in shaping technology adoption behavior ( Inegbedion et al., 2016; Schuster & Parkinson, 2022). Individuals who perceive DMHS as useful, beneficial, and appropriate are more likely to develop internal motivation to use these services. Within the context of the model of goal-directed behavior, this relationship highlights how cognitive appraisal serves as an important precursor to motivational activation. Positive shifts in consumers’ perceptions of DMHS are therefore likely to strengthen their desire to pursue digital mental health solutions. Although statistically significant, the structural effect size was relatively modest (f ^2^ = 0.025), suggesting that attitude contributes meaningfully but does not represent the dominant predictor of desire. The f-square effect size describes how much influence a variable has in a structural model ( Wong, 2019). This indicates that favorable perceptions alone may not be sufficient to generate strong motivational commitment unless supported by complementary emotional or social influences.

The relationship between subjective norm and desire was also statistically significant (β = 0.122, p = 0.013, t = 2.482), supporting H2. This finding underscores the importance of social influence in shaping digital mental health behavior. Encouragement and approval from family members, peers, or other significant social actors appear to reinforce individuals’ internal motivation to manage their mental health. The result aligns with evidence reported by Pretorius et al. (2019), which demonstrates that social endorsement increases online help-seeking behavior among younger adults. Mental health decision-making often occurs within broader interpersonal contexts, where support systems can reduce uncertainty and legitimize help-seeking behavior. Supportive familial and interpersonal relationships are essential for sustaining mental well-being ( Fusar-Poli et al., 2020). In collectivist social environments such as Indonesia, the influence of close relational networks may be particularly salient. However, similar to attitude, the effect size was relatively small (f ^2^ = 0.015), indicating that while subjective norm contributes to motivational formation, its explanatory influence remains limited when considered independently.

In contrast, perceived behavioral control did not significantly influence desire (β = 0.058, p = 0.246, t = 1.159), resulting in the rejection of H3. This result diverges from traditional MGB expectations, which propose that greater perceived control should strengthen motivational states ( Ajzen, 1991; Perugini & Bagozzi, 2001). The lack of significance here may indicate that confidence and resources alone are insufficient to generate emotional motivation for mental health management. This finding is the same as that found by Schuster et al. (2017) that PBC did not influence desire. Several explanations may account for this non-significant relationship. First, the sample consisted predominantly of young, digitally experienced, and highly educated respondents, which may have reduced variability in perceived control. When most individuals already feel capable of accessing digital services, perceived control may lose explanatory significance ( van Deursen & van Dijk, 2014). Second, prior technology adoption studies suggest that control-related predictors tend to diminish in importance among experienced users, as familiarity reduces perceived barriers ( Venkatesh et al., 2012). Third, mental-health help-seeking is heavily influenced by emotional factors and stigma, which can suppress motivational desire even when users feel practically capable of using DMHS ( Rickwood et al., 2005). This aligns with the notion that mental health–related behaviors often depend more on emotional and social reinforcement than on perceived capability ( Rosenstock, 1974). This interpretation is consistent with mental health adoption research suggesting that emotional readiness and psychological safety often outweigh functional competence in predicting engagement with digital mental health tools ( Torous et al., 2018). Taken together, these findings indicate that in digitally mature consumer segments, practical capability may represent a baseline condition rather than a motivating force.

The results further reveal that perceived severity significantly influenced desire to manage mental health (β = 0.138, p = 0.006, t = 2.737), supporting H4, but did not significantly affect behavioral intention to purchase DMHS (β = −0.006, p = 0.899), leading to the rejection of H5. This pattern suggests that recognizing the seriousness of mental health consequences increases psychological motivation for self-management but does not directly translate into purchasing intention. This finding is consistent with core assumptions of the health belief model, which posits that perceived seriousness functions as an internal motivational trigger for protective action ( Rosenstock, 1974; Becker, 1974). Consumers who recognize the potential consequences of unmanaged mental health—such as impaired productivity, deteriorating relationships, or reduced life satisfaction—appear more inclined to desire preventive action. This result is also consistent with prior digital health studies showing that perceived severity enhances motivational readiness ( Dou et al., 2017; Wei et al., 2021). However, the relatively small effect size (f ^2^ = 0.016) indicates that severity alone exerts limited structural influence. More importantly, its lack of direct impact on purchase intention suggests that awareness of risk is insufficient to produce market commitment. This disconnect may reflect practical concerns such as uncertainty regarding service effectiveness, privacy concerns, or reluctance to pay for mental health support despite acknowledging its importance.

A similar pattern emerged for perceived susceptibility, which significantly influenced desire (β = 0.156, p = 0.004, t = 2.908), supporting H6, but did not significantly affect behavioral intention (β = 0.044, p = 0.418, t = 0.810), resulting in rejection of H7. These findings indicate that personal perceptions of vulnerability stimulate psychological readiness to engage in self-care but are insufficient to directly motivate purchase behavior. This is consistent with the health belief model’s proposition that perceived susceptibility serves as an early motivational trigger for preventive cognition ( Champion & Skinner, 2008; Rosenstock, 1974). In digital mental health contexts, awareness of personal vulnerability may heighten concern and encourage contemplation of intervention. Consistent with prior studies, individuals who perceived higher health vulnerability were more willing to adopt mobile health technologies ( Dou et al., 2017; C.C. & Prathap, 2020). The effect size, however, remained modest (f ^2^ = 0.022), suggesting that susceptibility acts primarily as an initial psychological catalyst rather than a decisive behavioral determinant. The absence of a direct relationship with behavioral intention reinforces the notion that risk perception alone cannot fully explain digital service adoption.

Individuals may recognize vulnerability while simultaneously postponing action due to cost concerns, uncertainty, social stigma, or low confidence in digital therapeutic effectiveness. An interesting descriptive insight may help contextualize this result: 65.6% of respondents identified themselves as introverts. This characteristic may influence how threat perceptions translate into behavioral action. Introverted individuals may exhibit stronger internal awareness of psychological distress while remaining hesitant to engage in formal intervention pathways, including paid digital services. Different personalities can have different cognitive and behavioral characteristics ( Serenko, 2026). Although this explanation remains speculative and was not directly tested in the structural model, it offers a promising direction for future research examining personality as a potential moderator.

Finally, desire to manage mental health demonstrated the strongest and most statistically robust positive effect on behavioral intention to purchase DMHS (β = 0.179, p = 0.000, t = 3.818), thereby supporting H8. This result strongly reinforces the central proposition of the model of goal-directed behavior, which positions desire as the immediate motivational antecedent of intention ( Perugini & Bagozzi, 2001). The finding confirms that while evaluative and threat-based perceptions contribute to motivational formation, behavioral intention emerges primarily when these antecedents are translated into a genuine internal desire to act. This highlights desire as the key psychological mechanism bridging cognition and behavioral commitment in digital mental health adoption. The result is consistent with prior research across digital technology settings, which consistently identifies desire as a critical determinant of behavioral intention ( Thomas-Francois et al., 2023; Schuster & Parkinson, 2022). From a practical standpoint, this finding suggests that strategies aimed solely at increasing awareness of mental health risks may be insufficient. Digital mental health providers must instead focus on strengthening motivational desire through emotionally resonant communication, trust-building mechanisms, and value propositions that connect psychological need with actionable service engagement.

Overall, the findings demonstrate that DMHS adoption is shaped less by functional capability and direct threat perception than by the extent to which these factors activate internal motivational desire. This reinforces the relevance of integrating motivational and health belief perspectives when examining consumer behavior in emerging digital health markets.

## Conclusions and implications

This study examined consumer intention to adopt digital mental health services (DMHS) in Indonesia through an integrated framework combining the model of goal-directed behavior (MGB) and the health belief model (HBM). By positioning desire to manage mental health as the central motivational mechanism, the study provides empirical evidence regarding the psychological pathways that shape adoption decisions in the digital mental health context. The findings demonstrate that desire functions as the primary motivational channel through which evaluative and health-related perceptions are translated into behavioral intentions. Attitude toward purchasing DMHS and subjective norm were found to significantly enhance desire, indicating that both personal evaluations and social influence play meaningful roles in motivating mental health management through digital means. Similarly, perceived severity and perceived susceptibility significantly influenced desire, suggesting that individuals’ recognition of mental health risks contributes to motivational activation. However, perceived behavioral control did not exert a significant effect, indicating that functional capability or perceived ease of access alone is insufficient to stimulate psychological readiness in this context. Most notably, desire showed a strong positive relationship with behavioral intention, reinforcing its position as the most immediate predictor of DMHS adoption.

The robustness of the proposed framework is further supported by model evaluation indicators. The structural model demonstrated satisfactory explanatory capacity, meaningful predictive relevance, acceptable fit based on SRMR values, strong out-of-sample predictive performance, and generally stable structural relationships. The identification of several nonlinear effects additionally suggests that certain motivational influences may operate in more complex ways than linear models typically assume. Collectively, these results support the suitability of the integrated MGB–HBM framework for explaining consumer adoption behavior toward DMHS within emerging-market settings. However, although the model explained 28.3% of the variance in behavioural intention, substantial variance remains unexplained, suggesting that other psychological, social, technological, and contextual factors may also influence purchase intention.

### Theoretical contributions

This study contributes to literature in several important ways. First, it strengthens the theoretical relevance of the model of goal-directed behavior within digital mental health research by empirically confirming the central role of desire as a motivational determinant of adoption intention. Much of the existing DMHS literature has focused predominantly on technological acceptance variables or attitudinal predictors. The present findings extend this perspective by demonstrating that motivational readiness serves as the critical mechanism connecting cognitive evaluations to behavioral commitment. Second, the integration of health belief constructs into the MGB framework advances current understanding of digital health adoption. The results indicate that perceived severity and perceived susceptibility influence consumer intention indirectly through desire rather than through direct behavioral pathways. This finding provides a more refined explanation of mental health-related decision-making and offers a potential explanation for inconsistent findings reported in previous HBM-based digital health studies. Third, this study offers methodological contributions by nonlinearity analysis within PLS-SEM investigations. The observed curvilinear effects involving attitude, subjective norm, perceived behavioral control, and perceived susceptibility suggest that motivational processes may not develop in strictly proportional ways. This highlights the need for future consumer behavior models to account for more dynamic and nonlinear behavioral mechanisms. Finally, the Indonesian setting provides an important contextual contribution. Research on DMHS adoption remains heavily concentrated in developed economies. By focusing on Indonesia, this study broadens empirical understanding of digital mental health adoption within emerging-market contexts characterized by rapid digitalization, evolving mental health awareness, and culturally embedded social influences.

### Managerial implications

The findings offer several practical implications for DMHS providers, digital platform developers, and marketing practitioners. First, strategic efforts should focus on cultivating consumers’ desire to actively manage their mental health, as this construct emerged as the strongest determinant of purchase intention. This suggests that merely highlighting platform functionality or technical efficiency is unlikely to be sufficient. Instead, communication strategies should emphasize emotional resonance, personal well-being, empowerment, and self-care benefits. Second, marketing initiatives should capitalize on both attitudinal and social drivers. Messages that frame DMHS positively and communicate tangible psychological benefits are likely to strengthen consumer motivation. Likewise, endorsements from trusted social referents—including peers, family members, healthcare professionals, and mental health advocates—may prove particularly effective in encouraging adoption, especially within collectivist cultural settings. Third, although perceived severity and susceptibility contribute to motivational formation, these factors do not directly stimulate purchase intention. This indicates that risk-based communication should be applied carefully. Excessively fear-oriented messaging may increase awareness without encouraging action. A more effective approach would combine acknowledgment of mental health risks with supportive, solution-oriented narratives that present DMHS as accessible and empowering tools for self-management. Fourth, the limited explanatory role of perceived behavioral control suggests that usability improvements alone are insufficient to drive adoption. While intuitive interface design remains important, greater attention should be directed toward strengthening privacy assurance, trust, emotional engagement, and stigma-sensitive service design. Given the personal and vulnerable nature of mental health decision-making, these factors are likely to carry greater weight than technical convenience.

From a policy standpoint, the findings underscore the importance of addressing motivational and emotional dimensions when promoting digital mental health adoption. Public awareness campaigns should move beyond general informational messaging and instead emphasize psychological relevance, emotional preparedness, and culturally appropriate narratives that encourage proactive self-care. Government agencies, educational institutions, and healthcare organizations may also play a critical role by collaborating with DMHS providers to normalize digital mental health care. Such collaboration could include public education initiatives, integration of DMHS within broader healthcare delivery systems, and endorsement programs designed to increase trust and legitimacy. Reducing stigma remains especially important. Policies that promote mental health literacy and position digital mental health support as a legitimate and accessible component of modern healthcare may significantly strengthen both motivational desire and eventual adoption behavior. Overall, the findings suggest that successful expansion of the digital mental health sector requires not only technological readiness but also the cultivation of psychological readiness among consumers.

### Limitations and future research

Despite its contributions, this study has several limitations. First, the cross-sectional design limits causal inference. Future studies could employ longitudinal or experimental designs to examine changes in desire and intention over time. Second, the sample is skewed toward younger, digitally literate individuals, which may limit generalizability. Future research should include older populations and rural communities. Third, while this study focused on behavioral intention, future research should examine actual usage behavior, retention, and long-term engagement with DMHS. Finally, additional psychological variables such as stigma, trust, emotional distress, or personality traits may further enrich the explanatory power of the model.

## Ethical considerations

This study was approved by the Deputy Vice Chancellor (Research & Innovation), Professor Dr. Sandeep Poddar, on June 23, 2025, by the Research & Innovation Committee of Lincoln University College with the ethics approval number LUC/MKT/IND/SP/007/310. All participants were adults over 18 years of age. All data collected was anonymized to ensure participant privacy and confidentiality, and after explanation, respondents were asked to sign a consent form indicating their willingness to participate.

## Data Availability

Zenodo: The Indonesian DMHS (Digital Mental Health Service) Study. https://doi.org/10.5281/zenodo.19174087 ( Adam et al., 2026a). The project contains the following:
• DMHS study – raw data N = 462 Zenodo: The Indonesia DMHS Study Questionnaire. https://doi.org/10.5281/zenodo.18906360 ( Adam et al., 2026b). The project contains the following extended data:
• Questionnaire of the digital mental health services study Data are available under the terms of the Creative Commons Attribution 4.0 International.
