# Supplementary material for: Modeling Consumer Adoption of Digital Mental Health Services (DMHS) in Indonesia: The Role of Desire
Source: F1000Res. 2026 Oct 26;15:990. Originally published 2026 Jun 23. [Version 2] doi: 10.12688/f1000research.182672.2 (PMC13720293; doi:10.12688/f1000research.182672.2)
Supplement: Peer review history

# Reviewer report: Reviewer response for version 2

**DOI:** 10.5256/f1000research.213110.r533813

**Published:** 2026-11-05

**Authors:** Firmina Theresia Kora

**Affiliations:** 1 STIKES Wira Husada Yogyakarta, Yogyakarta, Indonesia

It has been revised according to the feedback regarding writing style, discussion, and other aspects. Thank you

Is the work clearly and accurately presented and does it cite the current literature?

Partly

If applicable, is the statistical analysis and its interpretation appropriate?

Yes

Are all the source data underlying the results available to ensure full reproducibility?

Yes

Is the study design appropriate and is the work technically sound?

Partly

Are the conclusions drawn adequately supported by the results?

Yes

Are sufficient details of methods and analysis provided to allow replication by others?

Yes

Reviewer Expertise:

Mental health

I confirm that I have read this submission and believe that I have an appropriate level of expertise to confirm that it is of an acceptable scientific standard.

# Reviewer report: Reviewer response for version 2

**DOI:** 10.5256/f1000research.213110.r533814

**Published:** 2026-10-30

**Authors:** Johan Budhiana

**Affiliations:** 1 Sekolah Tinggi Ilmu Kesehatan Sukabumi, Sukabumi, Indonesia

This article is good and acceptable.

Is the work clearly and accurately presented and does it cite the current literature?

Partly

If applicable, is the statistical analysis and its interpretation appropriate?

Yes

Are all the source data underlying the results available to ensure full reproducibility?

Yes

Is the study design appropriate and is the work technically sound?

Yes

Are the conclusions drawn adequately supported by the results?

Yes

Are sufficient details of methods and analysis provided to allow replication by others?

Yes

Reviewer Expertise:

Biostatistics, Public Health, Quantitative Research Methods

I confirm that I have read this submission and believe that I have an appropriate level of expertise to confirm that it is of an acceptable scientific standard.

# Reviewer report: Reviewer response for version 2

**DOI:** 10.5256/f1000research.213110.r533815

**Published:** 2026-10-29

**Authors:** andri alfi

**Affiliations:** 1 Swiss German University, Kota Tangerang, Indonesia

Thank you for the opportunity to review the revised manuscript. I am satisfied with the revisions and the authors’ responses to my previous comments. All points have been adequately addressed, and I have no further comments.

Is the work clearly and accurately presented and does it cite the current literature?

Partly

If applicable, is the statistical analysis and its interpretation appropriate?

Yes

Are all the source data underlying the results available to ensure full reproducibility?

Yes

Is the study design appropriate and is the work technically sound?

Yes

Are the conclusions drawn adequately supported by the results?

Yes

Are sufficient details of methods and analysis provided to allow replication by others?

Yes

Reviewer Expertise:

Technology Adoption and Technology Acceptance, Digital Transformation, Quantitative Research Methods and SEM-PLS

I confirm that I have read this submission and believe that I have an appropriate level of expertise to confirm that it is of an acceptable scientific standard.

# Reviewer report: Reviewer response for version 1

**DOI:** 10.5256/f1000research.201634.r524400

**Published:** 2026-10-14

**Authors:** Johan Budhiana

**Affiliations:** 1 Sekolah Tinggi Ilmu Kesehatan Sukabumi, Sukabumi, Indonesia

Overall, this manuscript addresses a relevant topic and has the potential to contribute to our understanding of consumers’ intentions to adopt digital mental health services in Indonesia. This study demonstrates a commendable effort to integrate the MGB and HBM, apply appropriate statistical analyses, and promote transparency by openly providing research data and instruments. Nevertheless, there are several aspects that could still be improved, particularly regarding the strengthening of the theoretical foundation, the accuracy of model evaluation interpretations, and the consistency between the analysis results and the conclusions. The following comments are provided to help enhance the manuscript’s argumentative rigor, methodological precision, and scientific contribution.

1. Overall, the manuscript is well-structured and supported by relevant literature. The integration of MGB and HBM offers an interesting perspective for understanding consumers’ intentions to adopt digital mental health services. However, the theoretical foundation linking the two models could be further developed. The authors are advised to strengthen the conceptual argument regarding the proposed relationship and to explain the added value of integrating the two models in explaining adoption behavior.
2. The research design and use of PLS-SEM are generally consistent with the research objectives, with evaluation procedures covering the measurement model, structural model, and predictive power. Nevertheless, there are several aspects that need to be considered in interpreting the results. The adequacy of the sample size of 462 respondents should not be based solely on the “10-fold rule” but should also be supported by considerations of statistical power. Additionally, the use of the GoF index to conclude the overall model fit needs to be reconsidered, given its limitations in evaluating PLS-SEM model specifications. The authors also need to ensure that claims regarding predictive power are consistently supported by PLS-Predict results and that conclusions remain focused on purchase intention, in line with the measured variables.
3. This manuscript provides a clear and detailed explanation of the research methodology, including the research design, population and sampling procedures, data collection, sample size, variable measurement, and statistical analysis. Overall, the research methods have been well explained and provide sufficient information to understand the research stages and support replication by other researchers.
4. The use of PLS-SEM is appropriate for the study’s objective of testing the relationships among variables within the integrated MGB–HBM model framework. The statistical analysis procedures have been adequately reported, and the interpretation of the results is generally consistent with the model testing outcomes. This study has also considered the evaluation of the structural model and its predictive power, thereby supporting an assessment of the proposed model’s strength.
5. The authors have demonstrated a strong commitment to research transparency and openness by providing raw data from 462 respondents as well as the research questionnaire through the Zenodo repository. The availability of both sources under an open license is a positive aspect that supports the verification of results and the reproducibility of the research. Overall, the data availability statement has been well presented.
6. The conclusions are generally consistent with the research findings, particularly regarding the role of desire as a motivational mechanism linking psychological factors and health perceptions to the intention to adopt digital mental health services. The authors have also integrated the results of structural equation modeling and model evaluation to strengthen the interpretation of the findings. As an area for improvement, the authors are advised to ensure that statements regarding the predictive power and model fit are fully consistent with the reported analysis results, and to consistently distinguish between the intention to adopt and the actual adoption of the service.

Is the work clearly and accurately presented and does it cite the current literature?

Partly

If applicable, is the statistical analysis and its interpretation appropriate?

Yes

Are all the source data underlying the results available to ensure full reproducibility?

Yes

Is the study design appropriate and is the work technically sound?

Yes

Are the conclusions drawn adequately supported by the results?

Yes

Are sufficient details of methods and analysis provided to allow replication by others?

Yes

Reviewer Expertise:

Biostatistics, Public Health, Quantitative Research Methods

I confirm that I have read this submission and believe that I have an appropriate level of expertise to confirm that it is of an acceptable scientific standard, however I have significant reservations, as outlined above.

# Reviewer report: Reviewer response for version 1

**DOI:** 10.5256/f1000research.201634.r524398

**Published:** 2026-10-09

**Authors:** andri alfi

**Affiliations:** 1 Swiss German University, Kota Tangerang, Indonesia

**Overall Assessment**

 This manuscript examines the factors that influence Indonesian consumers’ intention to purchase digital mental health services (DMHS) by combining the Model of Goal-Directed Behavior (MGB) with selected constructs from the Health Belief Model (HBM). The study focuses particularly on the role of desire as a motivational mechanism linking consumers’ perceptions and evaluations to their behavioral intention.

 The topic is relevant, particularly given the increasing availability of digital mental health services and the need to understand how consumers respond to these services. The manuscript also has a clear empirical structure. The research questions are generally understandable, the methodology is explained in sufficient detail, and the use of PLS-SEM is reasonable for examining the proposed relationships. The sample of 462 valid respondents also provides a useful basis for the analysis.

 However, the manuscript needs some revision before it is ready for indexing. My main concern is not research design, but rather the way the theoretical arguments and empirical findings are presented and interpreted. In several places, the manuscript moves from the statistical findings to broader conclusions without providing enough theoretical explanation or without making the limitations of the research design sufficiently clear.

 My recommendation is therefore Approved with reservations

 1. Is the work clearly and accurately presented and does it cite the current literature 

 The article provides a clear research background and presents the research objectives, methodology, findings, and conclusions in a logical manner. The authors draw on recent literature to develop the theoretical framework, particularly by integrating the Model of Goal-Directed Behavior (MGB) and the Health Belief Model (HBM). The introduction further refers to relevant studies on digital mental health services and users’ behavioral adoption.

 However, the theoretical justification for the proposed relationships could be developed further. While the authors introduce the MGB and HBM in the Introduction, the discussion of how the individual constructs are expected to influence desire and behavioral intention remains somewhat limited. The manuscript would be strengthened by providing a more explicit discussion of the theoretical reasoning behind each hypothesized relationship, supported by relevant empirical studies. This would help clarify why these relationships are expected and provide a stronger foundation for the proposed conceptual model. 

 2. Is the study design appropriate and is the work technically sound?

 The quantitative method is appropriate for examining the relationships between the behavioural constructs and adoption intention. The study use of PLS-SEM with Smart-PLS is suitable, considering the complexity of the research model and its focus on predictive relationships.

 However, some methodological aspects could be discussed further. Firstly, although the authors have acknowledged this limitation in the manuscript (Page 14), the cross-sectional design does not allow for strong conclusions about causal relationships. Secondly, the use of non-probability sampling, particularly purposive and snowball sampling, may have resulted in a sample dominated by younger, digitally literate, and urban respondents. Thus, this may limit the generalizability of the findings to a broader population. In addition, the authors should consider addressing this when interpreting their results.

 On the other hand, the authors could provide a clearer distinction between behavioral intention and actual adoption. Although the study focuses on consumer adoption of digital mental health services, measuring intention at a single point in time seems like does not reflect whether respondents will actually use these services. Clarifying this distinction would help ensure that the findings and conclusions remain consistent with what the research design and measurements can establish. Finally, the practical implications could be strengthened, particularly in explaining how the concept of desire can be applied in marketing and communication strategies. For instance, digital mental health service providers could focus on empowerment-oriented and privacy focused messages rather than relying mainly on fear based appeals. Highlighting users’ control over their personal information, confidentiality, convenience, and the potential benefits of these services could help build trust and encourage adoption. Providing more specific examples of these strategies would make the study's practical contribution clearer and more useful for service providers.

 3. Are sufficient details of methods and analysis provided to allow replication by others?

 The manuscript provides a clear and detailed explanation of the research methodology i.e., the population criteria, sampling strategy, data collection procedures, measurement scales, and statistical analysis. The authors also report 462 valid responses and provide sufficient information about the sampling process, questionnaire development, and measurement of the constructs. Overall, the methodological procedures are presented clearly and provide a good foundation for understanding how the study was conducted.

 4. If applicable, is the statistical analysis and its interpretation appropriate?

 The statistical methods used in the study are appropriate, and the authors have provided a comprehensive assessment of the measurement and structural models, including reliability, validity, VIF, R², Q², SRMR, and hypothesis testing. These analyses provide a sound basis for evaluating the proposed research model. However, the main issue is how the statistical results are interpreted. The authors should be careful not to interpret significant paths as evidence of causality, and they should avoid overstating the practical magnitude of the relationship between "Desire" and "behavioral intention".

 5. Are all the source data underlying the results available to ensure full reproducibility?

 The manuscript provides a clear data availability statement indicating that the underlying data are publicly available under *“The Indonesian DMHS Study (Digital Mental Health Service)”*. Thus, the questionnaire is provided as extended data, which allows other researchers to better understand and use this study as their study references or replicate the study. Furthermore, the data are made available under a Creative Commons Attribution 4.0 International (CC BY 4.0) license. Overall, the data availability information is clear and sufficient.

 6. Are the conclusions drawn adequately supported by the results?

 The conclusions are generally consistent with the research findings. The results show that the desire to manage mental health significantly influences behavioral intention, while perceived behavioral control and direct threat perceptions do not have significant effects. however, the conclusions should be narrowed in few places i.e., the study demonstrates relationship with behavioral intention, not actual consumer adoption or purchasing behavior. On the other hand, The authors should also acknowledge that the model explains 28.3% of the variance in behavioral intention, meaning that other factors remain important. Acknowledging this would help the authors present a more balanced interpretation of their findings and identify opportunities for future research.

Is the work clearly and accurately presented and does it cite the current literature?

Partly

If applicable, is the statistical analysis and its interpretation appropriate?

Yes

Are all the source data underlying the results available to ensure full reproducibility?

Yes

Is the study design appropriate and is the work technically sound?

Yes

Are the conclusions drawn adequately supported by the results?

Yes

Are sufficient details of methods and analysis provided to allow replication by others?

Yes

Reviewer Expertise:

Technology Adoption and Technology Acceptance, Digital Transformation, Quantitative Research Methods and SEM-PLS

I confirm that I have read this submission and believe that I have an appropriate level of expertise to confirm that it is of an acceptable scientific standard, however I have significant reservations, as outlined above.

# Response

**Published:** 2026-10-18

**Authors:** Mochammad Riyadh Rizky Adam

**Affiliations:** Business & Management, Lincoln University College, Petaling Jaya, Selangor, Malaysia

Thank you for the reviews. We have addressed the concerns. Some of it was already in the paper.

# Reviewer report: Reviewer response for version 1

**DOI:** 10.5256/f1000research.201634.r500914

**Published:** 2026-08-07

**Authors:** Firmina Theresia Kora

**Affiliations:** 1 STIKES Wira Husada Yogyakarta, Yogyakarta, Indonesia

-
  - The primary dependent construct in the structural modal is the behavioral intention to purchase. However, throughout the abstract and Conclusion sections, the authors frequently refer to "consumer adoption" or "purchasing behavior" as if actual usage/transaction was empirically tested.
  - In the *Methods* section (Page 5), the authors state: *"Attitude toward purchasing DMHS: six items adapted..."* and *"Desire: three items adapted..."*. However, the exact wording of all 35 operational indicators is not printed directly in the text body or supplementary tables. Append a comprehensive measurement scale table (showing item codes, full wording in English and Bahasa Indonesia, outer loadings, and cross-loadings) directly within the main manuscript or formal appendix for reader clarity.
  - *Page 2 (Grant Information):* There is repetitive phrasing regarding grant funding ("Publication funding for this article was provided by..." followed immediately by "The funders had no role in study design..."). Please merge into standard concise wording.
  - Expand practical recommendations for digital health providers on **how** to operationalize "Desire" in marketing communications (e.g., shifting from fear-based health warnings to empowerment and privacy-first messaging).

Is the work clearly and accurately presented and does it cite the current literature?

Partly

If applicable, is the statistical analysis and its interpretation appropriate?

Yes

Are all the source data underlying the results available to ensure full reproducibility?

Yes

Is the study design appropriate and is the work technically sound?

Partly

Are the conclusions drawn adequately supported by the results?

Yes

Are sufficient details of methods and analysis provided to allow replication by others?

Yes

Reviewer Expertise:

Mental health

I confirm that I have read this submission and believe that I have an appropriate level of expertise to confirm that it is of an acceptable scientific standard, however I have significant reservations, as outlined above.

# Response

**Published:** 2026-10-31

**Authors:** Mochammad Riyadh Rizky Adam

**Affiliations:** Business & Management, Lincoln University College, Petaling Jaya, Selangor, Malaysia

Thank you for the reviews. Appreciate.
